# Evaluation of KH560 on properties and environmental effects of electrolytic manganese slag-based cementitious materials

**DOI:** 10.1016/j.isci.2025.112449

**Published:** 2025-04-16

**Authors:** Ying Zhou, Yue Wang, Daikuan Huang, Yang Cao, Dabin Zhang

**Affiliations:** 1 School of Mechanical Engineering, Guizhou University, Guiyang 550025, China; 2 Guizhou Research and Designing Institute of Environmental Sciences, Guiyang 550081, China

**Keywords:** Chemistry, Environmental science, Materials science

## Abstract

KH560-modified electrolytic manganese slag (EMS)-based cementitious material (EP-K) was successfully prepared in this study through aqueous solution polymerization for use in deep grouting to repair leaking landfills. EP-K with 3 vol % KH560 exhibits higher compressive strength (125.14% higher) and lower permeability (61.29% lower) than before modification. The concentration of contaminants leached in deionized water meets safety standards. Fourier transform infrared (FTIR) and scanning electron microscope (SEM) analyses showed that the epoxy groups in KH560 reacted with the –OH groups of sodium polyacrylate to form a C–O–C structure, which enhanced the densification of the cementitious network. Also, the siloxy groups reacted with EMS to form a Si–O–Si linkage, which reduced holes and cracks. The integrated pollution indicator evaluation method was combined with the weight assignment technique to enhance evaluation efficiency and accuracy, advancing research on the performance and environmental impact of cementitious impermeable materials in solid waste landfills.

## Highlights

• KH560 increases mechanical strength of cementitious material
• KH560 reduces water penetration
• KH560 boosts curing of contaminants
• Chemical bonds and physical entanglement ensure the success of modification

## Graphical abstract

**Figure:**
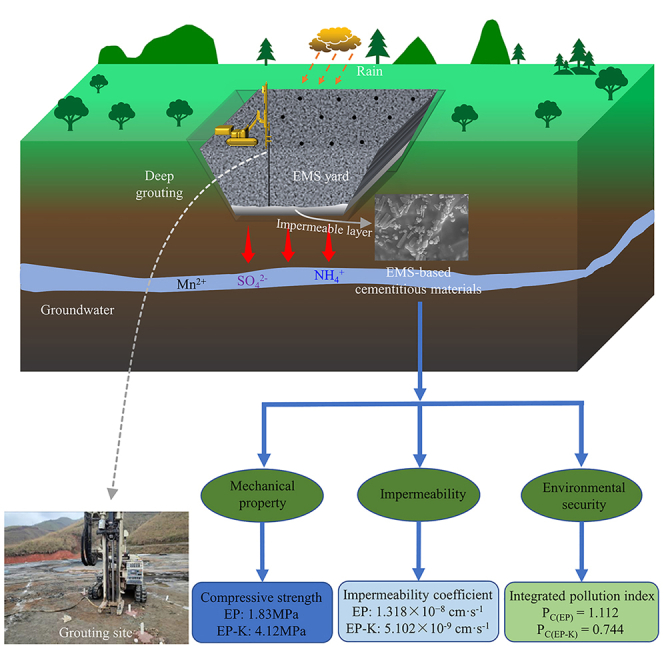

## Introduction

Industrial waste, such as electrolytic manganese slag (EMS), is often arbitrarily piled up in open dumps. Water-soluble components like Mn^2+^, NH_3_-N, and SO_4_^2−^ from these dumps can cause transboundary water pollution through surface water and groundwater, seriously jeopardizing the ecological environment.^1^^,^^2^ To address the pollution issues associated with seepage dumps, *in situ* deep grouting technology can be effectively utilized to establish an impervious barrier.^3^ Materials such as cement and bentonite are widely used in the remediation of groundwater or contaminant seepage from slag dumps.^4^ Studies have shown^5^^,^^6^^,^^7^ that the impermeable barrier constructed using cement and bentonite will destroy the original crystal structure of bentonite under a sulfate environment so that the cations between the crystal layers are replaced, thus affecting the stability of the crystal structure and resulting in the impermeability failing.

Polyacrylates are widely used for seepage prevention in underground engineering due to their excellent chemical resistance and toughness.^8^^,^^9^^,^^10^ However, single-component polyacrylate cementitious materials suffer from poor mechanical properties, susceptibility to water absorption and swelling, and cracking, which limit their application in solid waste landfill seepage prevention.^11^ Ghasemzadeh,^12^ Bahranifard,^13^ and Sandomierski^14^ successfully improved the mechanical properties of cementitious composites by adding sodium dodecyl sulfate, styrene, and silicon dioxide to polyacrylates. To prevent cracks in polyacrylate cementitious composites, Majeed et al.^15^ added inorganic filler nano-montmorillonite to polyacrylates, effectively inhibiting water absorption and swelling, thereby preventing the formation of cracks. Nonetheless, cementitious composites containing inorganic fillers may experience differences in swelling upon water absorption, leading to detachment of the filler from the matrix, which in turn affects their modification effect or performance.^16^ Additionally, Ezenkwa^17^ added organic rice hulls to polyacrylates, which prevented crack propagation and improved the compressive properties of the cementitious composite by about 20%. Li et al.^18^ also observed that organic fibers in the polymer matrix could fill and connect the pores, helping to reduce crack formation. However, the non-uniform distribution of organic fillers may lead to localized aggregation and internal voids, which can affect the mechanical strength of the material. Moreover, in humid environments, organic fillers may absorb water, swell, or dissolve, leading to the failure of the modification effect.

Silane coupling agents are organosilicon modifiers with amphoteric groups that optimize the interaction between inorganic particles and organic matrices, enhance the mechanical properties of materials, promote interfacial bonding, and improve material stability.^19^ Ye et al.^20^ showed that the use of silane coupling agent KH550 could improve the interfacial compatibility between polyurethane and charcoal ash slag and enhance the mechanical properties of the cementitious composite. Xing^21^ added the silane coupling agent KH570 to slag particles, which reduced the elastic modulus of the cementitious composite, enhanced ductility, and reduced surface cracks. Another study by Li^22^ showed that the addition of KH560 to the cementitious composite increased the tensile strength and elongation at the break by approximately 23.66% and 33.18%, respectively. Uzay,^23^ Liu,^24^ Li,^25^ and others further compared and analyzed the modes of action of aminosilane coupling agents KH550 and KH570 and the methoxysilane coupling agent KH560. They found that silane coupling agents containing amino groups were more hydrophilic in aqueous environments, while methoxyl group silane coupling agents demonstrated better adhesion and stability. These studies indicate that the addition of an appropriate amount of methoxyl group silane coupling agent can effectively inhibit the formation of cracks in cementitious composites and improve water stability. However, the mechanism by which silane coupling agents enhance interfacial strength and inhibit cracks in EMS-based cementitious materials still requires further in-depth study.

In this study, γ-(2,3-epoxypropoxy) propyltrimethoxysilane (KH560) was introduced into EMS and sodium polyacrylate (PAAS) via aqueous solution polymerization, successfully preparing the organosilicon-modified EMS-based cementitious composite (EP-K). The research analyzed the changes in compressive strength, corrosion resistance, permeability coefficient, and porosity of the cementitious composite. Molecular structure characterization techniques were used to analyze the changes in surface morphology and the content of the main elements of the cementitious composite, revealing the mechanisms underlying the enhancement of mechanical properties and the anti-seepage effects of KH560 on cementitious materials. Subsequently, changes in contaminant ion concentrations in the leachate were detected through cementitious composite immersion experiments. The integrated pollution index method, improved by the weight assignment method, was used to evaluate the water safety of the cementitious materials after curing and maintenance, taking into account typical contaminant types and their interactions. The study’s results provide theoretical support for solid waste landfill seepage grouting remediation, mine groundwater resource management, and environmental protection.

## Results and discussion

### Synthesis of EMS-based cementitious materials

**Figure 1:**
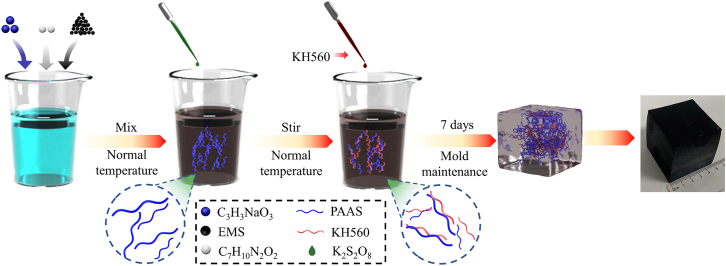
Preparation diagram of cementitious composite

EMS from a manganese slag reservoir in southwest China, with its composition detailed in Table 1, was sieved and dried. The EMS-based cementitious composite was then synthesized using an aqueous solution polymerization method, as illustrated in Figure 1. Sodium acrylate was used as the copolymerizing monomer, N, N-methylene diacrylamide as the cross-linking agent, and KH560 as the modifying agent. The amount of KH560 added was optimized based on the unconfined compressive strength of the cementitious composite. The compressive strength increased with the addition of KH560 but decreased after reaching a certain level. The most significant enhancement in compressive strength was observed when the volume doping of KH560 was 3%.

**Table 1:** Main components and content of EMS

Components	MnO	Al_2_O_3_	Fe_2_O_3_	SiO_2_	SO_3_	CaO	H_2_O
Content (wt %)	3∼4	3∼10	4∼8	25∼36	18∼27	8∼12	17∼27

### Characterization and performance tests

**Figure 2:**
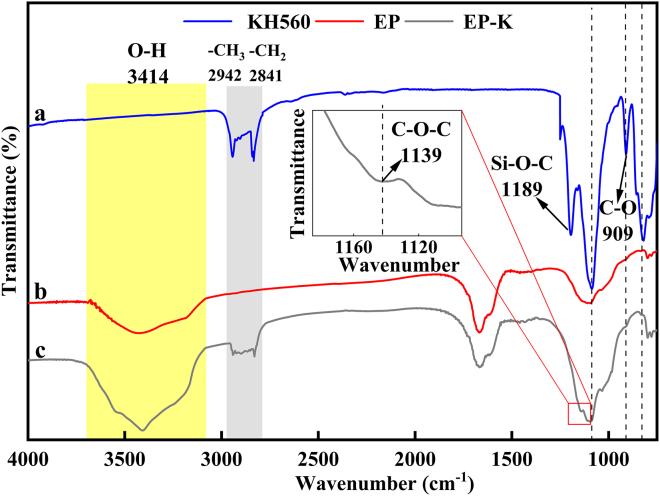
FTIR spectra of KH560, EP, and EP-K

Figure 2 shows the Fourier transform infrared (FTIR) spectra of KH560, the cementitious composite without KH560 (EP), and EP-K. In the spectrum of KH560, the stretching vibration peaks of C–O and Si–O–C bonds appeared at 909 and 1,189 cm^−1^, respectively, while the stretching vibration peaks of –CH_2_ and –CH_3_ were located at 2,841 and 2,942 cm^−1^, respectively. Compared with curve a, curve c shows the disappearance of the –CHOCH_2_ epoxy group at 909 cm^−1^ and the Si–O–C stretching vibration peak at 1,189 cm^−1^, as well as the appearance of the –CH_2_ and –CH_3_ stretching vibration peaks at 2,841 and 2,942 cm^−1^, respectively. This indicates that the epoxy group of KH560 chemically reacts with –OH on the carboxyl group of PAAS, resulting in successfully branched PAAS.^26^ Compared with curve b, curve c shows a significant increase in the peak of –OH stretching vibration at 3,414 cm^−1^, indicating that KH560 may form intermolecular hydrogen bonds with PAAS. Additionally, a new peak appears at 1,139 cm^−1^, suggesting that the silicone oxygen group Si–O–CH_3_ in KH560 was hydrolyzed to generate the silanol group Si–OH and that the epoxy group –CHOCH_2_ in the molecular structure of KH560 reacted with PAAS to form a C–O–C linked molecular chain structure.^27^

**Figure 3:**
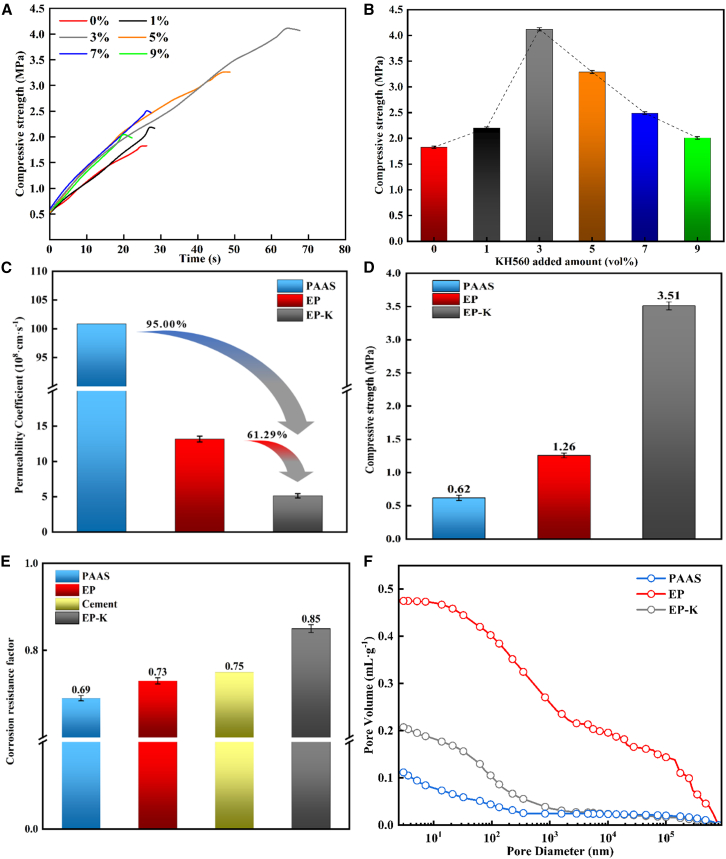
Performance testing experiments Unconfined compressive strength curve and ultimate compressive strength (A and B), permeability coefficient (C), ultimate compressive strength after corrosion and corrosion resistance coefficient (D and E), and cumulative pore volume (F). Data in (B), (C), (D), and (E) are represented as mean ± SEM.

During the modification process, we investigated the effect of adding different amounts of KH560 on the mechanical properties of EMS-based cementitious composite and assessed the mechanical properties of the cementitious composite using the unconfined compressive strength test. The test results are shown in Figure 3A indicates that, with the increase of time, the compressive strength of the cementitious composite exhibits a nonlinear rapid growth trend until reaching a rupture inflexion point. The longest compressive test time of the cementitious composite was obtained when the KH560 doping was 3 vol %. As shown in Figure 3B, the maximum compressive strength of the cementitious composite reaches its highest value at this inflexion point. The analysis revealed that the average unconfined compressive strength of the cementitious composite increased and then decreased with the increase of KH560, peaking at 3 vol %, with a value of 4.12 MPa. This represents a 125.14% increase compared to the cementitious composite without KH560. According to the experimental data and literature,^28^ this effect may be due to the physical entanglement of KH560 and PAAS molecular chains, which enhances the interfacial interaction force, makes the cementitious composite more compact, and thus improves the mechanical properties. When KH560 is added in too small an amount, there is less physical entanglement, and the intermolecular force within the cementitious composite is weaker, resulting in less noticeable improvement in mechanical properties. Conversely, when too much KH560 is added, the molecular chains become excessively entangled and piled up, which, due to steric hindrance effects, reduces the mechanical properties.

The permeability coefficients of the cementitious composite were obtained using the variable head test and calculated according to Equation 1 (see Figure 3C). The average permeability coefficient of EP-K with 3 vol % KH560 added was 5.102 × 10^−9^ cm s^−1^, while that of EP was 1.318 × 10^−8^ cm s^−1^. The average permeability coefficient of EP-K was 61.29% lower than that of EP. Compared with the standard value of 1 × 10^−7^ cm/s for type I in acrylate grouting material (JCT 2037-2010), it was reduced by 95.00%. This indicates that the addition of KH560 improved the impermeability of the cementitious composite. It is speculated that Si–OCH_3_, a siloxy group in the KH560 molecule, underwent a hydrolytic condensation reaction with free water in the EMS to form a Si–O–Si crosslinked polymer. This polymer filled the inter-particle pore space and increased the compactness of the cementitious composite, thereby enhancing its impermeability, which is in agreement with the results reported in the literature.^29^ Additionally, the hydrolysis of Si–OCH_3_ absorbed some of the free water on the surface of the EMS, reducing the amount of water infiltration in the system and improving the water stability of the cementitious composite.

The evaluation of the corrosion resistance of the cementitious composite needs to consider its compressive strength values before and after corrosion. According to the ultimate compressive strength of cementitious composite after sulfate corrosion (Figure 3D), the compressive strength of EP after corrosion is 1.26 MPa, which is 30.72% lower than its ultimate compressive strength of 1.82 MPa before corrosion. The corrosion resistance coefficient of EP is calculated to be 0.73 (Figure 3E). For EP-K doped with 3 vol % KH560, the maximum unconfined compressive strength after corrosion is 3.51 MPa, which is 14.86% lower than its ultimate compressive strength of 4.12 MPa before corrosion, resulting in a corrosion resistance coefficient of 0.85. This is significantly better than that of cement (0.75).^30^ It is speculated that KH560 accelerated the hydrolytic polymerization reaction of EMS in the salt solution containing SO_4_^2−^, forming a dense CaSO_4_·2H_2_O sediment layer, which impeded the penetration of SO_4_.^2–^.^31^

The pore structure analysis results shown in Figure 3F indicate that the cumulative pore volume of the EP-K cementitious composite supplemented with 3 vol % KH560 is significantly reduced to 0.207 mL g^−1^, a decrease of 56.42% compared to the EP cementitious composite (0.475 mL g^−1^). This demonstrates that KH560 significantly improved the microstructure of EMS-based cementing materials. It is concluded that the reaction of the Si–OH produced by the hydrolysis of KH560 with the hydroxyl groups on the surface of EMS weakens the van der Waals forces between particles, thereby inhibiting agglomeration and improving dispersion. As a result, PAAS molecules can be more effectively adsorbed on the surface of EMS particles to form a denser coating, ultimately reducing the porosity of the cementitious composite.^32^ This microstructural improvement is consistent with the improvement of the aforementioned macro-mechanical properties, further validating the role of KH560 as an effective modifier.

### Environmental assessment

**Figure 4:**
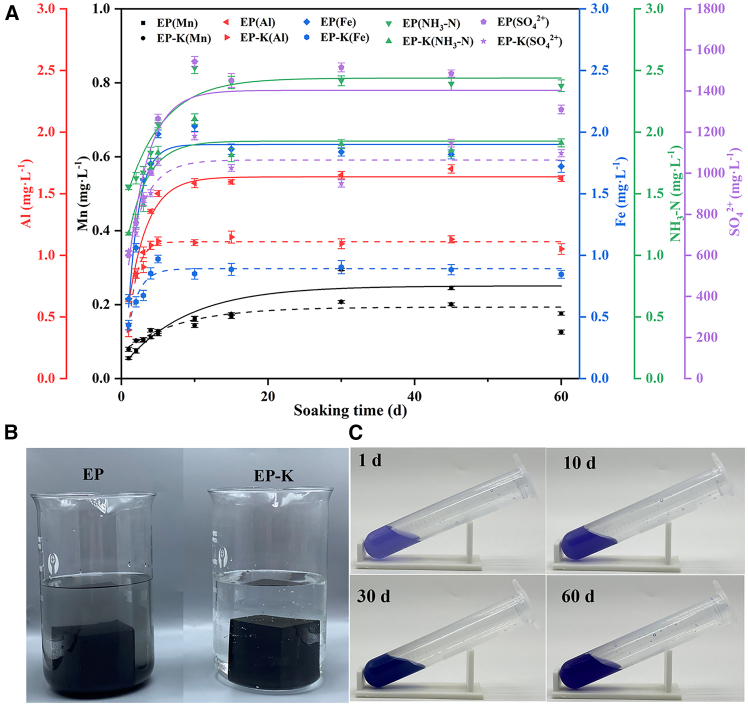
Contaminant leaching tests Leaching contaminant concentration fitting curve of EP and EP-K (A), leaching radiation change (B), and metal Mn color reaction (C). Data in (A) are represented as mean ± SEM.

Grouted impermeable barriers for solid waste landfills should ensure sufficient mechanical strength and seepage resistance while being environmentally friendly to avoid secondary pollution of groundwater. We focused on the leaching concentration changes of typical metal ions Mn, Al, Fe, NH_3_-N, and SO_4_^2−^ based on the contaminant types in the Groundwater Quality Standard (GB/T 14848-2017) and components of EMS (Table 1). Static leaching experiments with deionized water were carried out for EP and 3 vol % KH560-doped EP-K for 60 days. The experimental results are shown in Table S1. Based on the data from Table S1, a fitting curve for the change in contaminant leaching concentration was established using the method described in the literature,^33^ as shown in Figure 4A. In the early stage of the experiment (0–10 days), various ions precipitated rapidly, and the leaching concentration increased quickly. Among these, the metal ions Al and Fe rapidly reached a stable state after the 5th day, while the leaching concentrations of Mn ions, NH_3_-N, and SO_4_^2−^ continued to increase. In the middle and late stages of the experiment (10–60 days), the leaching concentrations of Al and Fe remained unchanged, while those of Mn, NH_3_-N, and SO_4_^2−^ stabilized after the 30th, 20th, and 15th day, respectively. It was demonstrated that the leaching rate of metal ions Al and Fe was significantly faster than that of Mn ions, NH_3_-N, and SO_4_^2−^ during the leaching process of cementitious composite, which is in agreement with the conclusion of the literature,^34^ as Mn, NH_3_-N, and SO_4_^2−^ are more soluble in water than Al and Fe.^35^ After 60 days, the EP leachate became turbid, while the EP-K leachate remained clear, as shown in Figure 4B. Table S1 data show that the leaching concentration of each ion in the EP-K leach solution at the end of the experiment was lower than that in the EP leach solution, indicating that KH560 can improve the solidification and adsorption of contaminations by the cementitious composite. This improvement is likely due to the reaction between KH560 and PAAS, forming a dense cementitious network structure that adsorbs contaminants.^36^ To observe the leaching of Mn ions, we took a small amount of EP-K leach solution to observe the color change (Figure 4C). In an alkaline environment, catechol violet forms a stable blue complex with Mn^2+^.^37^ After 1 day of immersion, the lighter color of the leachate indicates a low concentration of Mn^2+^; after 10 days, the darker color suggests an increase in the leaching concentration of Mn^2+^; after 30 and 60 days, the color does not change significantly, indicating that the leaching concentration of Mn^2+^ stabilizes in the mid-and late stages. This observation is consistent with the fitting results shown in Figure 4A. The correlation coefficients (*R*^*2*^) of the leaching concentration fitting equations, as reported in Table S2, ranged from 0.839 to 0.980, indicating a strong correlation between the leaching concentration of contaminants and time.

**Figure 5:**
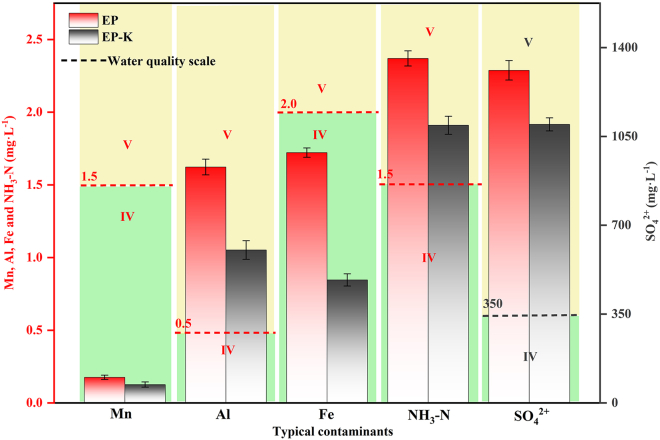
Water quality classes for single factor pollution index Data are represented as mean ± SEM.

Based on the Groundwater Quality Standard (GB/T 14848-2017), this study conducted a systematic safety assessment of EP and EP-K leachates. The experimental results (Figure 5) showed that the EP-K samples with KH560 addition had significant adsorption effects on typical metal ions, with the average leaching concentrations of Mn, Al, and Fe ions reduced by 38.2% compared with the control group. In contrast, the leaching concentrations of NH_3_-N and SO_4_^2−^ were only reduced by 19.4% and 16.2%, respectively. This difference indicates that the addition of KH560 significantly enhanced the adsorption capacity of EP-K for metal ions.

Further analysis (Figure 5 and Table S3) showed that the ionic concentrations of Mn and Fe in the EP and EP-K leachates met the Class-IV water quality standard, while the ionic concentrations of Al, NH_3_-N, and SO_4_^2−^ reached the Class-V water quality standard. Based on the principle of comprehensive evaluation, the water quality of the leachate was determined to be Class-V. Through the analytic hierarchy process (AHP) method, the experimental data were substituted into Equations 4, 5, 6, 7, and 8 to calculate the feature vectors, and the weight set *Q* was obtained, as shown in Table 2. After the consistency test, the consistency ratio (*CR* = 0.0022) was less than 0.1, indicating that the judgment matrix has good consistency and the weight allocation results are reliable. Finally, the integrated pollution index (*P*_*C*_) of EP and EP-K leachates was calculated as 1.112 and 0.744, respectively, according to Equation 3, confirming that the water quality safety of EP-K was significantly better than that of EP.

**Table 2:** Weight distribution result

Infusion	Contaminant weight				
	Mn	Al	Fe	NH_3_-H	SO_4_^2-^
*Q*(EP&EP-K)	0.577	0.236	0.110	0.048	0.029

### Mechanism of KH560 modification

**Figure 6:**
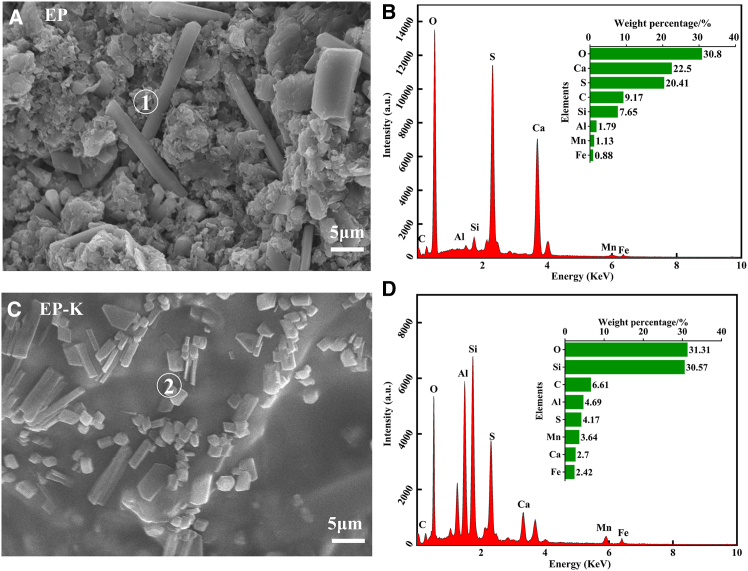
SEM-EDS analysis results SEM images of EP and EP-K (A and C) and EDS spectra (B and D) Scale bar: 5 μm.

Scanning electron microscope (SEM) morphology observations of the cementitious composite are presented in Figure 6. Figure 6A shows that the EP surface is loose, with rod-like material and agglomerated flocculated particles cross-stacked, displaying an obvious pore structure. The agglomerated flocculated particles were mainly composed of SiO_2_, Al_2_O_3_, CaO, and iron-containing manganese compounds in manganese slag, while the rod-shaped particles were primarily CaSO_4_.^38^^,^^39^ In contrast, the surface of EP-K in Figure 6C was smooth and flat, with the rods and agglomerated flocculated particles fused with the colloid, showing no obvious pores. EDS spectral analysis of the solidified bodies in Figures 6B and 6D revealed that the atomic contents of Al, Mn, and Fe elements in EP-K were significantly increased compared to EP, while the contents of C, S, and Ca elements were significantly decreased. This could be attributed to the ionized acrylic anionic group –COO^−^ from the PAAS slurry coordinating with the metal ions in the EMS during diffusion, contributing to the formation of a multi-crosslinked structure polymer. Additionally, KH560 promoted the hydrolysis polymerization reaction of EMS to form Si–O–Si linked polymers. These two polymers were physically entangled, enhancing the intermolecular forces of the cementitious material and resulting in a denser surface.

**Figure 7:**
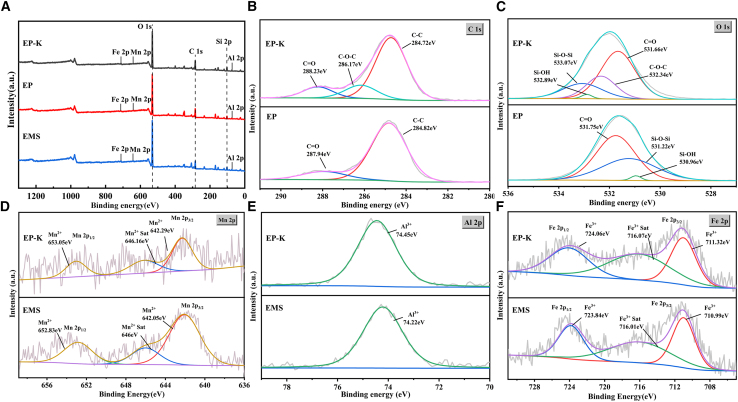
XPS analysis results Full-scale XPS spectra of EMS, EP, and EP-K (A) and the high-resolution XPS spectra of EMS, EP, and EP-K for C 1s of (B), O 1s (C), Mn 2p (D), Al 2p (E), and Fe 2p (F)

The results of the XPS spectral analysis are shown in Figure 7. The full spectrum scans (Figure 7A) confirmed the presence of major characteristic elements (C, O, Si, Mn, Al, and Fe) in the EMS, EP, and EP-K samples. In the C 1s high-resolution spectra (Figure 7B), both EP and EP-K showed characteristic peaks of PAAS, with C=O bonds at 287.94 and 288.23 eV, respectively, and C–C bonds at 284.82 and 284.72 eV, respectively. Notably, EP-K exhibited a characteristic C–O–C peak at 284.72 eV, which, in combination with FTIR analysis results, indicated that KH560 was successfully grafted onto PAAS via an epoxy ring-opening reaction.

O 1s spectral analysis (Figure 7C) revealed significant differences in the Si–O–Si and Si–OH binding energies of EP-K and EP, which were 1.15 and 1.93 eV, respectively, attributed to the hydrolysis reaction of KH560.^40^ Additionally, the difference in the binding energies of the C=O characteristic peaks of EP and EP-K further suggests that the –COO^−^ group plays a key role in the EMS-based cementitious system, participating in metal ion coordination and epoxy ring-opening reactions.^41^^,^^42^ The fine spectral analysis of Mn, Fe, and Al elements (Figures 7D–7F) showed that the binding energies of Mn^2+^ (642.05 and 652.83 eV), Fe^3+^ (710.99 and 723.84 eV), and Al^3+^ (74.22 eV) were significantly lower in EP-K compared to the EMS samples. Combined with the results of the SEM-EDS analyses, this confirms that the –COO^−^ group of PAAS has a stronger coordination ability with metal ions such as Mn^2+^, Al^3+^, and Fe^3+^, thereby enhancing the binding strength of the polymer with a multiple crosslinked structure.^43^

**Figure 8:**
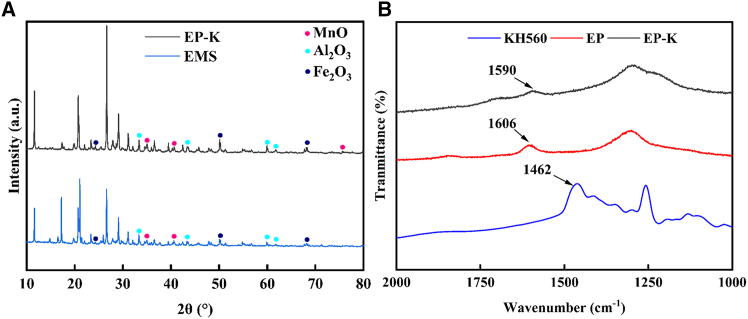
XRD and Raman analysis results XRD patterns of EMS and EP-K (A) and Roman spectra of KH560, EP, and EP-K (B)

The XRD pattern shown in Figure 8A indicates that the phase composition of EP-K is similar to that of EMS. After the addition of PAAS and KH560, the diffraction peaks of MnO, Fe_2_O_3_, and Al_2_O_3_ are enhanced, and a broadening of the diffraction peaks is observed. This may be due to the adsorption of metal ions such as Mn^2+^, Al^3+^, and Fe^3+^ by PAAS, which inhibits the crystallization of these metal oxides and thereby increases the content of amorphous phases.^44^ Additionally, KH560 improved the dispersion of EMS particles, allowing PAAS to be more uniformly adsorbed on the particle surface, further promoting the formation of amorphous phases.

Raman spectral analysis results are shown in Figure 8B. The characteristic spectrum of KH560 shows a flexion vibration peak of –CH_3_ on the silane group at 1,462 cm^−1^. The EP sample exhibited the O=C stretching vibration absorption peak in the PAAS carboxyl group at 1,606 cm^−1^. Notably, the characteristic peak of the carboxyl group in EP-K was redshifted to 1,590 cm^−1^, which was attributed to the formation of hydrogen bonds between the Si–OH hydrolyzed by KH560 and the –COO^−^ of PAAS (HO–C…O–H).^45^^,^^46^ In addition, the characteristic absorption peak of –CH_3_ was not observed in the EP-K spectra, indicating that KH560 had been completely hydrolyzed to Si–OH with a hydrolysis rate of 98.5%.

**Figure 9:**
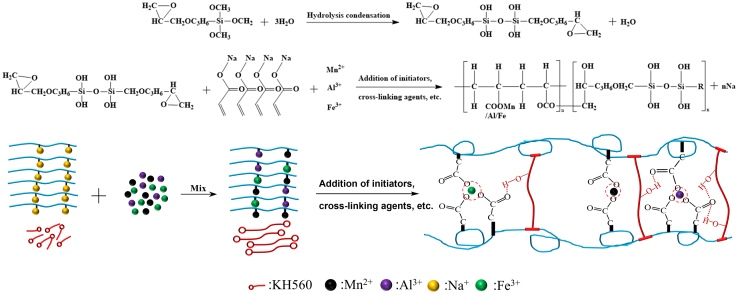
EMS, KH560, and acrylic acid polymerization reaction (R is –CH_3_OCH_2_CH_2_O(CH_2_)_3_)

**Figure 10:**
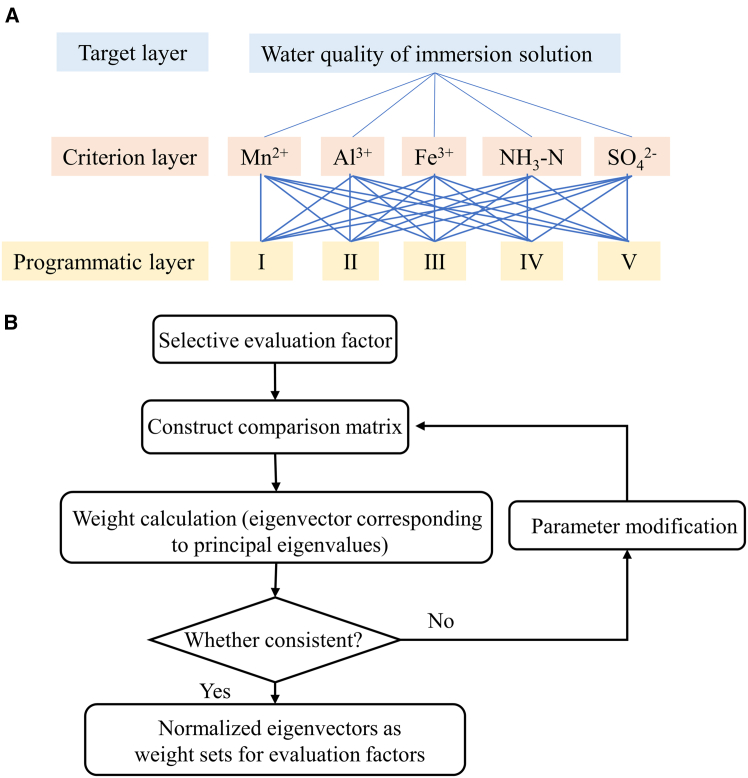
Weight allocation scheme based on the AHP method Hierarchy (A) and weight building step (B) in AHP

The mechanical enhancement mechanism and the anti-seepage effect mechanism of EP-K are illustrated in Figure 9. The siloxy group Si–OCH_3_ in KH560 undergoes a hydrolytic condensation reaction with free water on the surface of the EMS to form polymers containing Si–OH and Si–O–Si groups, with Si–OH hydrogen-bonded to carboxylate groups. Additionally, the epoxy functional group –CHOCH_2_ in KH560 undergoes a ring-opening polymerization reaction with the Mn^2+^-, Al^3+^-, and Fe^3+^-containing colloid to form a C–O–C bonded polymer. The physical entanglement network, supported by the chemical bonds and hydrogen bonds of the two polymers, enhances the interaction forces between the molecules of the cementitious composite, reduces cracks and pores, makes the surface of the cementitious composite denser and smoother, and reduces the water gap channels, thereby mitigating the impact on water quality. To demonstrate the proposed water quality evaluation method in detail, Figure 10 illustrates the structure of the AHP method and the process of constructing contaminant weights. Table 3 displays the magnitude of the quantitative values for the pairwise comparison of contaminants. Detailed information can be found in the STAR Methods section.

**Table 3:** The 1–9 point scale used for the pairwise comparisons

Relative importance	Scale	Relative importance	Scale
Equally important	1	equally important	1
Moderately important	3	moderately important	1/3
Strongly important	5	strongly important	1/5
Very strongly important	7	very strongly important	1/7
Extremely important	9	extremely important	1/9
Intermediate values	2,4,6,8	intermediate values	1/2,1/4,1/6,1/8

### Conclusions

In this study, the KH560-modified EMS-based composite adhesive EP-K was successfully prepared by aqueous polymerization, resulting in a cementitious composite with a multiple crosslinked network structure. At a KH560 content of 3 vol %, the unconfined compressive strength of EP-K reached 4.12 MPa, which is 125.14% higher than that of EP without KH560 modification. The permeability coefficient of EP-K was 5.102 × 10^−9^ cm s^−1^, 61.29% lower than that of EP and significantly lower than the type I permeability coefficient value of 1 × 10^−7^ cm s^−1^ specified in the standard for acrylic grouting materials (JCT 2037-2010), representing a reduction of 95.00%. Its corrosion resistance was also significantly better than that of cement. Using the improved integrated pollution index evaluation method, the ion concentrations of typical contaminants Mn, Fe, Al, NH_3_-N, and SO_4_^2−^ in the EP-K leaching solution were found to meet water quality safety requirements. This improvement is due to the reaction of the silica group Si–OCH_3_ in KH560 with free water on the surface of EMS, forming long-chain polymers with Si–OH and Si–O–Si structures. Additionally, the organic functional group –CHOCH_2_ and PAAS containing Mn^2+^, Al^3+^, and Fe^3+^ undergo an epoxy ring-opening reaction to form a C–O–C bond polymer. These polymer molecular chains enhance the densification of the cementitious material through chemical bonding and physical entanglement, reducing the water gap channels and making the surface flat and dense. This, in turn, improves the mechanical properties, corrosion resistance, seepage resistance, and curing effect on contaminants.

### Limitations of the study

Due to equipment limitations, this study did not include performance testing of EMS-based cementitious materials from the deeper portions of the grouted impermeable layer at the project site, and the evaluation of groundwater contaminant types was not comprehensive. Future work should aim to address these limitations by conducting relevant performance tests on cementitious materials at engineering sites and expanding monitoring to include other contaminants such as Ni, Cd, Cu, and Sb. This approach will broaden the practical applications of EMS in various scientific and industrial fields.

## Resource availability

### Lead contact

Requests for further information and resources and reagents should be directed to and will be fulfilled by the lead contact, Yue Wang (ywang9@gzu.edu.cn).

### Materials availability

This study did not generate new unique reagents. All chemicals were obtained from commercial resources and used as received.

### Data and code availability

Data: all data reported in this paper will be shared by the lead contact upon request.

Code: this study does not generate a new code.

Additional information: any additional information required to reanalyze the data reported in this study is available from the lead contact upon request.

## Acknowledgments

This work was supported by the 10.13039/501100012166National Key Research and Development Program of China (no. 2022YFC3705000) and 10.13039/501100018555Science and Technology Program of Guizhou Province (no. [2021]485 and no. [2023]346).

## Author contributions

Y.Z.: data curation and writing – original draft; Y.W.: supervision, writing – review and editing, and funding acquisition; D.H.: funding acquisition and investigation; Y.C.: investigation; D.Z.: funding acquisition and supervision.

## Declaration of interests

The authors declare no competing interests.

## STAR★Methods

### Key resources table

**Table:** 

REAGENT or RESOURCE	SOURCE	IDENTIFIER
**Chemicals, peptides, and recombinant proteins**		

EMS	Guizhou, China	N/A
C_3_H_3_NaO_3_	Tianjin Komeo Chemical Reagent Co., Ltd.	CAS: 7446-81-3
C_7_H_10_N_2_O_2_	Tianjin Komeo Chemical Reagent Co., Ltd.	CAS: 110-26-9
K_2_S_2_O_8_	Shenzhen Fulin Instrument Technology Co., Ltd.	CAS: 7727-21-1
C_9_H_20_O_5_Si	Nantong Feiyu Bio-Tech Co., Ltd.	CAS: 2530-83-8

**Software and Algorithms**		

OriginPro9.1	OriginLab	https://www.originlab.com/

### Experimental model and study participant details

This study does not use experimental methods typical in the life sciences.

### Method details

#### Synthesis of EMS-based cementitious materials

First, a certain amount of EMS was screened through a 6-mesh square-hole sieve and then dried in a constant temperature oven at 40°C for 24 hours. Subsequently, 144 g of EMS, 28.8 g of sodium acrylate (the masses of EMS and sodium acrylate were calculated based on a molar ratio of 3:1), and 0.6 g of N, N-methylene diacrylamide were mixed. Then, 95 mL of deionized water was added and stirred to form a mixed solution. To this mixed solution, 1 mL of initiator potassium persulfate was added, followed by an appropriate amount of KH560 solution. The mixture was stirred for 5 minutes at room temperature. Finally, it was poured into a 50×50×50 mm^3^ cube mould and maintained for 7 days to prepare EP-K sample blocks. Based on the volume of the mixed solution, 1, 3, 5, 7, and 9 vol% of KH560 were added, and the above steps were repeated to prepare each group of sample blocks.

#### Characterization

The FTIR spectra were measured by a Fourier Transform Infrared Spectrometer (FTIR-850) in the range of 400–4000 cm^-1^. The microscopic morphology of the samples was observed using a Feiner benchtop electron microscope energy spectrometer all-in-one (Phenom ProX) (JEOL JMS-6390), and the elemental atom percentages were analyzed using an EDS energy spectrum analyzer. X-ray photoelectron spectroscopy (XPS, Thermo Scientific K-Alpha) was used to detect characteristic elements in the sample at a depth of about 5 to 10 nm. The crystal structure of the sample was analyzed by X-ray diffractometer (XRD, Rigaku SmartLab SE), using a Cu-Kα radiation source (incidence angle λ=0.15406 nm) and a scanning angle range of 10°-80°. The laser Raman tests were performed using the Laser Raman Spectrometer (Horiba JY HR-800) with a wavelength of 514.5 nm and a power of 100 MW.

#### Unconfined compressive strength

The DYE-300S type automatic constant stress testing machine was used to test the unconfined compressive strength of the cementitious composite test block, with a loading speed of 100 N·s^-1^. The compressive strength-time curve was recorded from the start of the test until an inflex-ion point appeared after the cementitious composite was destroyed. The test was then terminated, and the ultimate compressive strength value was recorded.

#### Impermeability

The permeability coefficient was determined based on the variable head test standard in the civil engineering test methods standard (GB/T50123-2019). The cementitious composite was placed in the ring knife of the permeability meter and maintained for 1 day. The ring knife containing the cementitious composite was then placed into the permeability test container. The cross-sectional diameter of the glass tube was 0.6 cm, the cross-sectional diameter of the cementitious composite was 6.2 cm, and the length of the specimen was 4.0 cm. The initial head height was set at 90.0 cm, and the height of the water level was recorded after 24 hours of the test. By measuring the water head height before and after the experiment, the sample permeability coefficient Kr was calculated according to the empirical Equation 1.

(Equation 1) $$K_{r}=\frac{a\cdot L}{A\cdot \left(t_{1}-t_{0}\right)}\ln\left(\frac{h_{1}}{h_{0}}\right)$$

Where *K*_*r*_ is the permeability coefficient, cm·s^-1^; a is the cross-sectional area of the glass tube, cm^2^; *L* is the thickness of the sample, cm; *t*_*0*_ is the initial time, s; *t*_*1*_ is the termination time, s; *A* is the cross-sectional area of the specimen, cm^2^; *h*_*0*_ and *h*_*1*_ are the height of the head at the start and the head at the termination, cm, respectively.

#### Corrosion resistance

According to the standard for long-term performance and durability test methods of ordinary concrete (GB/T 50082-2009) for sulfate erosion resistance, the corrosion time was 96 hours. The compressive strength of the cementitious composite after the completion of corrosion was tested using the DYE-300S testing machine. The corrosion resistance was evaluated by comparing the compressive strength values of the cementitious composite before and after corrosion, and the corrosion resistance coefficient was calculated.

#### Porosity

The samples, maintained for 7 days, were cut into cubes with dimensions of 10 × 10 × 10 mm^3^. To fully remove the water from the samples and avoid the effect of high temperature on the sample structure, the cut samples were soaked in anhydrous ethanol for 7 days, then dried in a constant temperature oven at 40°C for 24 hours. The porosity of the cementitious composite was measured using a mercury intrusion porosimeter (AutoPore IV 9500), with a pore size range of 3.02 nm to 8.19 × 10^5^ nm and a pressure range of 0.23 to 6000 psi. Each sample was measured three times, and the average value was taken. The tests were conducted at room temperature, between 15-25°C.

#### Leaching concentration of contaminants

In the leaching experiments, EP and EP-K cementitious composite were placed in separate beakers and subjected to static leaching treatment at a solid-liquid ratio of 1:10. At 1, 2, 3, 4, 5, 10, 15, 30, 45, and 60 days, 15 mL of aqueous samples were removed for testing, and an equal volume of deionized water was added to the beaker to maintain the solid-liquid ratio. The water samples were then filtered through 0.45 μm aqueous filtration membranes, acidified with 65% concentrated nitric acid, and analyzed by inductively coupled plasma mass spectrometry (ICP-MS) for the leaching content of typical metal ions such as Mn, Al, and Fe. The leaching content of NH_3_-N and SO_4_^2-^ was measured using a T6 Xin Yue visible spectrophotometer. Each water sample test was repeated three times. To efficiently and accurately evaluate water quality, the integrated pollution index method was improved by considering typical pollutant types and their interactions and reassigning weights to typical metal ions, NH_3_-N, and SO_4_^2-^ using the Analytic Hierarchy Process (AHP) method.^47^ The AHP method primarily relies on the expertise of professionals in relevant fields. The relative importance of the evaluation factors is compared to construct a judgment matrix, and the weight of each evaluation factor is ultimately determined. Experts consider the toxicity, concentration, and impact on human health of the contaminants to make subjective judgments.

The formula is:

(Equation 2) $$P_{i}=\frac{C_{i}}{C_{Oi}}$$

(Equation 3) $$P_{c}=\sum_{i=1}^{N}\omega_{i}P_{i}$$

Where *P*_*i*_ is the single-factor pollution index; *C*_*i*_ is the measured concentration of the *i*th evaluation factor, mg·L^-1^; *C*_*oi*_ is the evaluation standard concentration of the *i*th evaluation factor, mg·L^-1^; *P*_*c*_ is the integrated pollution index; *N* is the number of contaminations participating in the evaluation; *ω*_*i*_ is the weight of the *i*th evaluation factor, determined by AHP method.

According to the Groundwater Quality Standard (GB/T 14848-2017), groundwater quality is divided into five categories. In this study, a hierarchy is constructed based on this standard, and the hierarchy and weights are determined through the process shown in Figures 10A and 10B. The 9-scale method was used as a comparison scale (Table 3) to measure the relative importance between the evaluation indicators.

Construct an expert judgment matrix A with scale *a*_*ij*_:

(Equation 4) $$A=[\begin{array}{cccc} a_{11} & a_{12} & \cdots & a_{1j}\\ a_{21} & a_{22} & \cdots & a_{2j}\\ \vdots & \vdots & & \vdots \\ a_{i1} & a_{i2} & \ldots & a_{ij} \end{array}]$$

Where *a*_*ij*_ indicates the degree of importance of evaluation factor *i* relative to evaluation factor *j* according to the experts. The specific range of values for *a*_*ij*_ is shown in Table 3, where 1 means that parameter *i* and parameter *j* have the same priority, and 9 means that parameter *i* is extremely more important than parameter *j*. Conversely, if one parameter has a lower priority than the other, the inverse value (the second column of Table 3) is taken to reflect the degree of importance.

The maximum characteristic root *λ*_*max*_ of the judgment matrix *A* is directly calculated to obtain the eigenvector *W*. The formula is shown in Equation 5:

(Equation 5) $$AW=\lambda_{\max}W$$

Where *W=* (*b*_*1*_, *b*_*2*_, …, *b*_*n*_).

To further determine the reasonableness of the weighting indicators, a consistency test of the judgment matrix is required. This can be calculated according to Equations 6 and 7:

(Equation 6) $$CI=\left(\lambda_{\max}-n\right)/\left(n-1\right)$$

(Equation 7) CR=CI/RI

Where *CR* is consistency ratio, *CI* is the consistency index, *RI* is the average random consistency index, and *n* is the order of the judgment matrix.

In the study, *n*=5, so *RI*=1.12.^48^ When *CR*<0.1, the consistency test is passed; otherwise, further correction of the judgment matrix is needed. If the consistency test is passed, the eigenvector *W=* (*b*_*1*_, *b*_*2*_, …, *b*_*n*_) is normalized according to Equation 8:

(Equation 8) $$\omega_{i}=b_{i}/\sum_{j=1}^{n}b_{i}$$

Calculate *Q*= (*ω*_*1*_, *ω*_*2*,_ …, *ω*_*n*_), which is the set of evaluation factor weights.

The colour change of water samples was observed using catechol violet as a manganese ion colourant. The pH value of the filtered leachate was adjusted to 8–10 with 40% pure NaOH solution. Catechol violet colourant was then added to the leachate, and the mixture was shaken well to observe the colour change.

### Quantification and statistical analysis

This study does not include statistical analysis or quantification.
