# Supplementary material for: Evaluation of KH560 on properties and environmental effects of electrolytic manganese slag-based cementitious materials
Source: iScience. 2025 Apr 16;28(5):112449. doi: 10.1016/j.isci.2025.112449 (PMC12059693; doi:10.1016/j.isci.2025.112449)
Supplement: Document S1. Tables S1–S3 [file mmc1.pdf]

iScience, Volume *28*

Supplemental information

Evaluation of KH560 on properties and environmental effects of electrolytic manganese slag-based cementitious materials

Ying Zhou, Yue Wang, Daikuan Huang, Yang Cao, and Dabin Zhang

Table S1

t/d	c (Mn) /(mg·L^{-1})		c (Al) /(mg·L^{-1})		c (Fe) /(mg·L^{-1})		c (NH_3-N) /(mg·L^{-1})		c (SO_4^{2+}) /(mg·L^{-1})	
	EP	EP-K	EP	EP-K	EP	EP-K	EP	EP-K	EP	EP-K
1	0.055	0.079	0.638	0.395	0.645	0.434	1.553	1.177	600.0	620.9
2	0.075	0.102	0.835	0.852	1.062	0.623	1.629	1.281	756.3	710.5
3	0.104	0.105	1.025	0.905	1.614	0.675	1.676	1.416	882.1	862.2
4	0.112	0.130	1.358	1.082	1.743	0.854	1.835	1.706	1002.5	902.6
5	0.121	0.127	1.501	1.115	1.986	0.970	2.064	1.833	1266.3	1056.3
10	0.161	0.143	1.586	1.103	2.050	0.851	2.523	2.106	1543.8	1181.3
15	0.168	0.174	1.596	1.149	1.862	0.886	2.407	1.813	1451.9	1025.1
30	0.295	0.207	1.651	1.094	1.840	0.904	2.414	1.903	1515.5	950.4
45	0.245	0.201	1.702	1.131	1.820	0.884	2.394	1.858	1485.8	1146.5
60	0.176	0.126	1.623	1.052	1.722	0.846	2.376	1.914	1310.4	1097.4

Table S2

Ions	Soak	Function	R^2
Mn	EP	$y=-0.22\times exp(-x/8.68)+0.25$	0.839
	EP-K	$y=-0.12\times exp(-x/7.84)+0.19$	0.877
Al	EP	$y=-1.51\times \exp(-x/2.6)+1.64$	0.980
	EP-K	$y=-1.72\times exp(-x/1.12)+1.11$	0.958
Fe	EP	$y=-2.58\times exp(-x/1.49)+1.90$	0.928
	EP-K	$y=-0.93\times exp(-x/1.49)+0.89$	0.867
NH_3-N	EP	$y=-1.11\times exp(-x/4.93)+2.44$	0.955
	EP-K	$y=-1.08\times exp(-x/2.74)+1.92$	0.964
SO_4^{2+}	EP	$y=-1225.95\times exp(-x/2.72)+1403.38$	0.896
	EP-K	$y=-675.38\times exp(-x/2.58)+1064.45$	0.919

Table S3

Contaminants		Mn	Al	Fe	NH_3-N	SO_4^{2+}
Soak /(mg·L^{-1})	EP	0.176	1.623	1.722	2.371	1310.4
	EP-K	0.126	1.052	0.846	1.914	1097.4
Class-IV Water /(mg·L^{-1})		≤1.5	≤0.5	≤2	≤1.5	≤350
Class-V Water /(mg·L^{-1})		>1.5	>0.5	>2	>1.5	>350
